# Supplementary material for: Mendelian randomization does not support serum calcium in prostate cancer risk
Source: Cancer Causes Control. 2018 Oct 10;29(11):1073–80. doi: 10.1007/s10552-018-1081-5 (PMC6245088; doi:10.1007/s10552-018-1081-5)
Supplement: Supplementary file 2 — Supplementary material 2 (DOCX 13 KB) [file 10552_2018_1081_MOESM2_ESM.docx]

Supplementary Table 1. Mendelian randomization derived causal effects of a 0.5 mg/dL increase in serum calcium on overall and advanced prostate cancer using a multi-allelic instrument in PRACTICAL with iterative removal of one SNP from instrument at a time

| **SNP removed** | **Overall Prostate cancer**  **OR (95% CI)** | **Advanced Prostate cancer**  **OR (95% CI)** |
| --- | --- | --- |
| rs17251221 | 0.77 (0.43-1.40) | 1.30 (0.47-3.62) |
| rs10491003 | 0.85 (0.62-1.17) | 0.94 (0.49-1.81) |
| rs7481584 | 0.84 (0.59-1.19) | 0.96 (0.50-1.86) |
| rs7336933 | 0.80 (0.58-1.09) | 0.94 (0.48-1.81) |
| rs1570669 | 0.85 (0.51-1.18) | 0.98 (0.50-1.90) |

OR: Odds Ratio, 95% CI: 95% Confidence Interval. EA reflects the allele that increases serum calcium levels. OR (95% CI) represents the exponential increase in odds for each 0.5 mg/dL increase in serum calcium
